# Ileal Gastrointestinal Stromal Tumor as a Rare Cause of Gastrointestinal Bleed: A Case Report and Brief Review of the Literature

**DOI:** 10.7759/cureus.22856

**Published:** 2022-03-04

**Authors:** Adnan S Khan

**Affiliations:** 1 Internal Medicine, Thomas Jefferson University Hospital, Philadelphia, USA

**Keywords:** small bowel mass, ileum, gi bleed, tumor, gist

## Abstract

Gastrointestinal stromal tumors (GISTs) are rare, slowly growing submucosal tumors in the gastrointestinal tract. Due to its indolent nature, GIST can go undetected for a long period of time. Symptomatic patients will typically develop abdominal pain, abdominal fullness, nausea, vomiting, and loss of appetite. However, most commonly and recognizable, patients will have blood in their stool due to rapid bleeding from the tumor. We report a case of a late diagnosis of GIST in the ileum with a favorable outcome.

## Introduction

Gastrointestinal stromal tumors (GIST) are mesenchymal tumors of the gastrointestinal tract that usually express either KIT or PDGFRA [1]. Due to its slow-growing nature, many patients are asymptomatic. However, once symptomatic, GIST can lead to life-threatening complications. In our case, we aim to discuss an unusual location of GIST and its unique presentation.

## Case presentation

A 59-year-old female with an insignificant medical history was presented with syncope and melena from an outside hospital. Her hemoglobin level was found to be 5.5. She received 10 units of packed red blood cells, 2 units of fresh frozen plasma, and 1 unit of platelets. During her stay in the hospital, she underwent an EGD/colonoscopy which showed no evidence of active bleeding. However, the patient's melena persisted. She was transferred to a tertiary hospital for further management.

**Figure 1:**
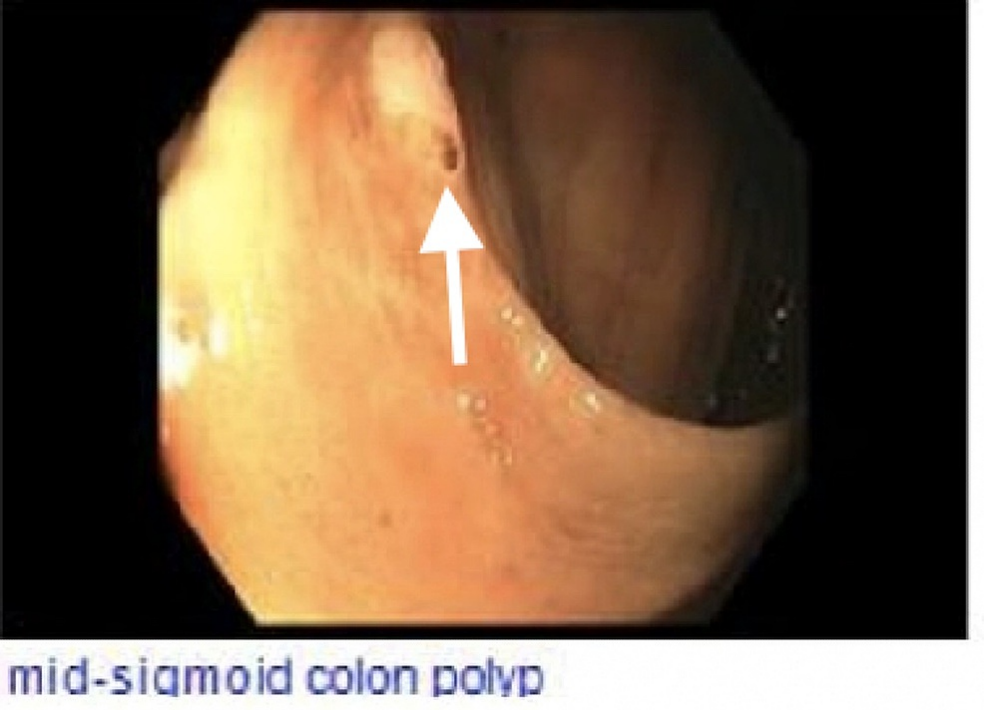
Mid-sigmoid colonoscopy White arrow shows diminutive polyp

**Figure 2:**
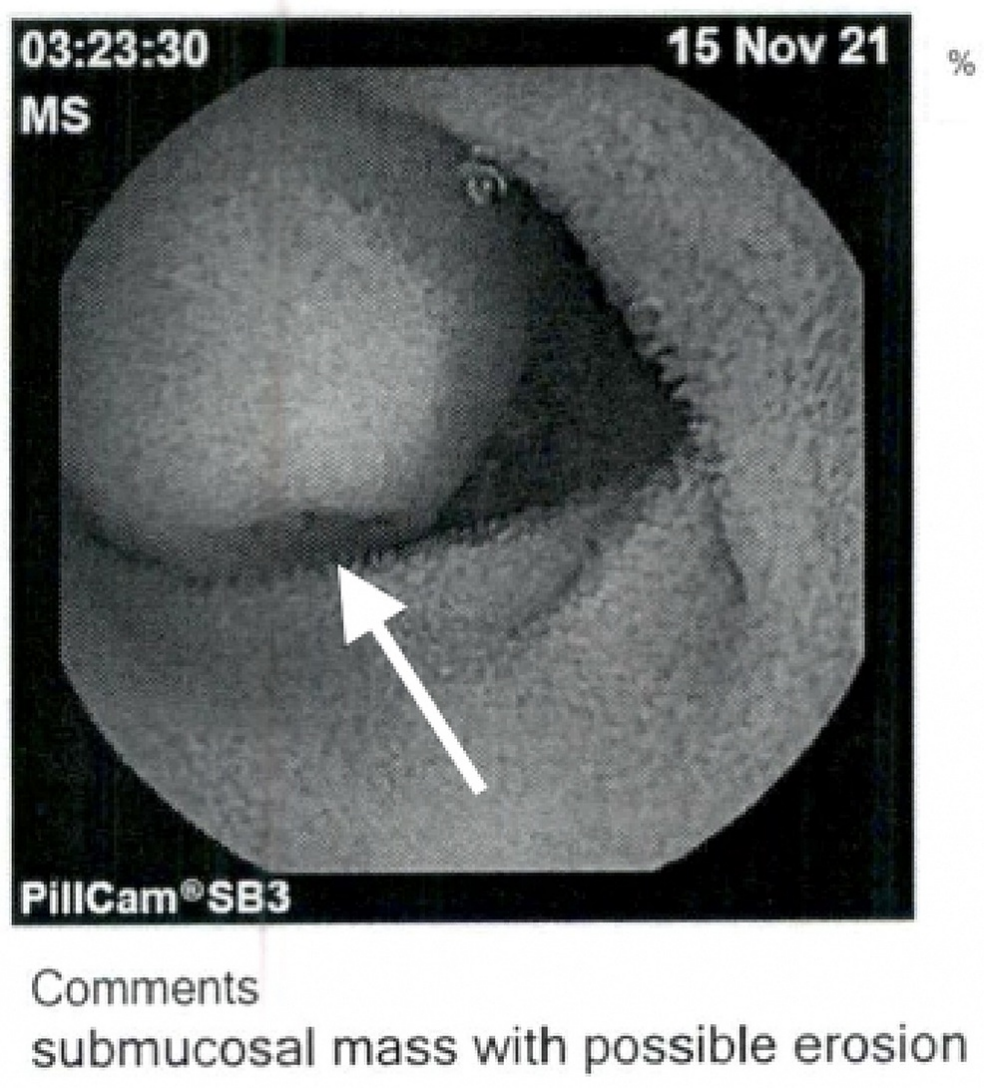
Capsule study White arrow shows submucosal mass with possible erosion

On arrival, she underwent an EGD/colonoscopy with a capsule study. The EGD showed a normal esophagus, stomach, and duodenum with no active source of bleeding. The colonoscopy showed a single sessile polyp of diminutive appearance in sigmoid colon status post polypectomy (Figure 1). The capsule study showed a possible submucosal mass in the mid-small bowel with central ulceration (Figure 2). The pathology of the sigmoid colon polyp showed tubular adenoma and adjacent colonic mucosa with melanosis coli.

**Figure 3:**
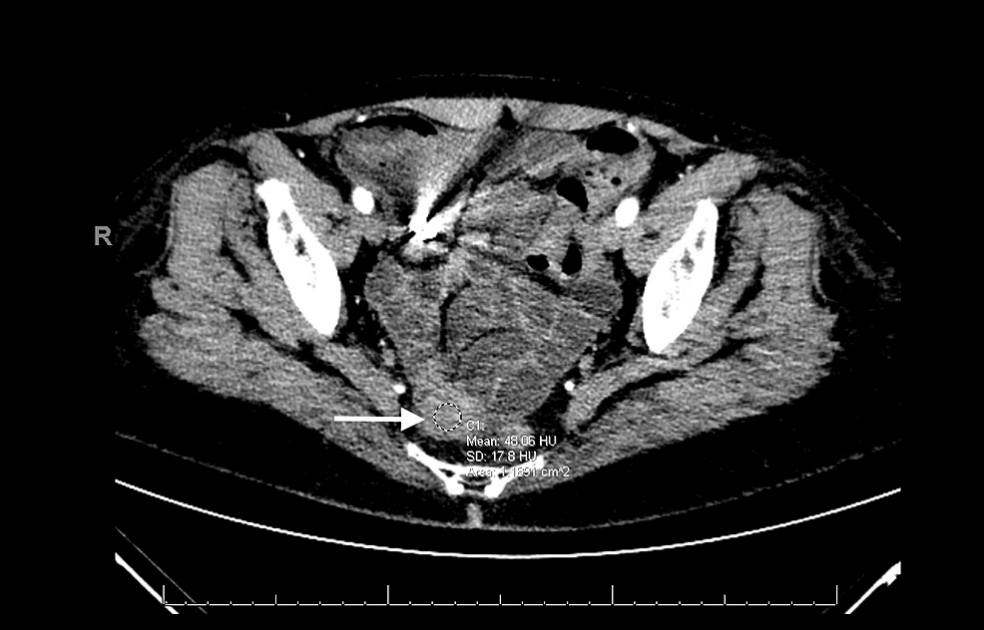
CTA abdomen and pelvis White arrow shows mid-ileal 3.3 × 2.6 × 2.7 cm^3^ mass favoring a GIST

The patient underwent a repeat CT angiogram of the abdomen and pelvis which showed mesenteric vasculature with no findings of ischemic bowel but did show a mid-ileal 3.3 × 2.6 × 2.7 cm^3^ mass favoring a GIST (Figure 3).

Laparoscopic small bowel resection was performed after consulting with the surgical team. The pathology of the specimen showed a 3.5 cm gastrointestinal stromal tumor arising from the ileum (not involving the ileocecal valve) with biomarkers positive for DOG1 and C-KIT (exon 9). The patient was discharged after resection and post-operative hemoglobin stabilization.

With no recurrence of GI bleeding at the one-month follow-up, the patient was advised to be closely monitored with no adjuvant imatinib. The patient was classified as low risk of recurrence given her KIT mutation on exon 9.

## Discussion

GISTs are classified morphologically as spindle (70%), epithelioid (20%), or mixed (10%) tumors that express either KIT or PDGFRA [2]. GISTs are slow-growing tumors that sometimes go undetected. The most common localization of GISTs is in the stomach (50-60%), followed by the small intestines (20-25%) and the rectum (5%) [3,4].

Symptomatic patients will typically develop abdominal pain, abdominal fullness, nausea, vomiting, and loss of appetite. However, patients can frequently present with GI bleeding. A systematic literature search was performed, which included 12 articles with a total of 2781 patients with GIST. The overall survival of patients with GIST-related GI bleeding was worse than that of non-GI bleeding. The study explained that aging factors and the location of GIST in the small intestine can increase the risk of GI bleeding in patients with GIST [4].

Current studies indicate that GIST originates from interstitial cells of Cajal (ICC) that proliferate in the myenteric plexus of the gastrointestinal tract [4,5]. ICCs (which express cKIT and DOG1 and variably express CD34) have recently been identified as pacemaker cells for gastrointestinal motor activity, initiating and maintaining peristalsis [4,5]. Prognosis depends on tumor size, mitotic rate, and site of origin [6].

Affected individuals with no family history of GIST typically have only one tumor, known as sporadic GIST. However, individuals with a family history of GISTs are classified as familial GISTs. Most people diagnosed with a GIST are older than 50, but it rarely affects people younger than 40 [7]. Rarely, GIST may be part of a genetic syndrome. The following genetic syndromes have been linked to GIST: neurofibromatosis type 1 (NF1) and the Carney triad (the combination of GISTs, paragangliomas, and pulmonary chondromas) [8,9]. A rare form of GIST, called succinate dehydrogenase (SDH)-deficient GIST, tends to occur in childhood or young adulthood and affects females more commonly than males [10,11].

If the tumor is smaller than 2 cm and not causing symptoms, the patient will be kept on surveillance due to its favorable prognosis [12]. Surgery is recommended only if cancer has not metastasized or spread to other areas of the body. A less invasive surgery, laparoscopy, is usually used for small GISTs that measure more than 2 cm but less than 5 cm. Invasive surgery may be an option for tumors larger than 5 cm. Depending on its location, partial gastrectomy, partial intestine resection, or abdominoperineal resection can be considered [13,14].

Targeted therapy can disrupt the mutated genetic markers. PDGFR is among the targets of many FDA-approved multikinase inhibitors. PDGFRA mutations in GIST typically involve exons 12 and 18 [15]. Imatinib, FDA approved, is a targeted treatment for KIT-positive unresectable or metastatic GIST. KIT exon 11 mutant GIST yields significantly higher response rates to imatinib and has longer overall survival than those with KIT exon 9 mutant [16,17]. Sunitinib, a multitarget TKI, was approved as a second-line treatment for advanced GIST after imatinib failure [18]. Regorafenib, a multitarget TKI, is a competitive inhibitor of the ATP-binding site for KIT, which has been approved as a third-line treatment of advanced GIST after progression on imatinib and sunitinib [19]. Most recently, Ripretinib received FDA approval on May 15, 2020, for the treatment of adult patients with advanced GIST who have received prior treatment with three or more kinase inhibitors, including imatinib [20].

## Conclusions

Gastrointestinal bleeding harbors a broad differential with the potential for oversight by GIST. However, due to its indolent nature, it is often diagnosed when patients develop symptomatic complications. Prognosis depends upon tumor size, mitotic rate, and site of origin. Prognosis will help guide medical decisions on which treatment will be most beneficial. Early intervention is imperative to minimize the risk of patient deterioration. Early detection of symptomatic GIST provided high-quality care in avoiding severe complications in our case.
